# Subchronic infusion of the product of inflammation prostaglandin J2 models sporadic Parkinson's disease in mice

**DOI:** 10.1186/1742-2094-6-18

**Published:** 2009-07-25

**Authors:** Sha-Ron Pierre, Marijke AM Lemmens, Maria E Figueiredo-Pereira

**Affiliations:** 1 Department of Biological Sciences, Hunter College, City University of New York, New York, N.Y. 10065, USA; 2 Department of Psychiatry and Neuropsychology, Division of Cellular Neuroscience, Maastricht University, 6200 MD Maastricht, The Netherlands

## Abstract

**Background:**

Chronic neuroinflammation is implicated in Parkinson's disease (PD). Inflammation involves the activation of microglia and astrocytes that release high levels of prostaglandins. There is a profound gap in our understanding of how cyclooxygenases and their prostaglandin products redirect cellular events to promote PD neurodegeneration. The major prostaglandin in the mammalian brain is prostaglandin D2, which readily undergoes spontaneous dehydration to generate the bioactive cyclopentenone prostaglandins of the J2 series. These J2 prostaglandins are highly reactive and neurotoxic products of inflammation shown in cellular models to impair the ubiquitin/proteasome pathway and cause the accumulation of ubiquitinated proteins. PD is a disorder that exhibits accumulation of ubiquitinated proteins in neuronal inclusions (Lewy bodies). The role of J2 prostaglandins in promoting PD neurodegeneration has not been investigated under *in vivo *conditions.

**Methods:**

We addressed the neurodegenerative and behavioral effects of the administration of prostaglandin J2 (PGJ2) simultaneously into the *substantia nigra*/*striatum *of adult male FVB mice by subchronic microinjections. One group received unilateral injections of DMSO (vehicle, n = 6) and three groups received PGJ2 [3.4 μg or 6.7 μg (n = 6 per group) or 16.7 μg (n = 5)] per injection. Immunohistochemical and behavioral analyses were applied to assess the effects of the subchronic PGJ2 microinfusions.

**Results:**

Immunohistochemical analysis demonstrated a PGJ2 dose-dependent significant and selective loss of dopaminergic neurons in the *substantia nigra *while the GABAergic neurons were spared. PGJ2 also triggered formation of aggregates immunoreactive for ubiquitin and α-synuclein in the spared dopaminergic neurons. Moreover, PGJ2 infusion caused a massive microglia and astrocyte activation that could initiate a deleterious cascade leading to self-sustained progressive neurodegeneration. The PGJ2-treated mice also exhibited locomotor and posture impairment.

**Conclusion:**

Our studies establish the first model of inflammation in which administration of an endogenous highly reactive product of inflammation, PGJ2, recapitulates key aspects of PD. Our novel PGJ2-induced PD model strongly supports the view that localized and chronic production of highly reactive and neurotoxic prostaglandins, such as PGJ2, in the CNS could be an integral component of inflammation triggered by insults evoked by physical, chemical or microbial stimuli and thus establishes a link between neuroinflammation and PD neurodegeneration.

## Background

There is increasing recognition that neuroinflammation is a major factor in the pathogenesis of neurodegenerative disorders such as Alzheimer's [1] and Parkinson's (PD) [2,3]. In particular, evidence suggests that prior occurrence of inflammation in the brain, due to either brain injury or infectious agents, may play a key role in PD pathogenesis [4]. It is proposed that a self-perpetuating cycle of inflammatory processes involving brain immune cells (microglia and astrocytes) may drive the slow progression of the neurodegenerative process. The neuroinflammation hypothesis for PD is supported by different kinds of evidence (reviewed in [5]) such as *(1) *increased numbers of astrocytes and microglia in affected brain areas as well as continued presence of activated microglia in the *substantia nigra*; *(2) *DNA polymorphisms in cytokines modify PD risk, in particular the age of onset; *(3) *protection by non-steroidal anti-inflammatory drugs (NSAIDs) although this is highly controversial, and *(4) *most *in vivo *and *in vitro *models of PD support involvement of inflammatory mechanisms. Many of the animal models for idiopathic PD are produced by neurotoxins, the four most popular being 6-hydroxydopamine (6-OHDA), MPTP (1-methyl 4-phenyl 1,2,3,6-tetrahydropyridine), rotenone and paraquat [6]. All of these animal models are associated with signs of neuroinflammation in the *substantia nigra*, supporting the view that inflammation and in particular glial cells including astrocytes and microglia, play a central role in PD [5].

It is thought that the dopaminergic neurons in the *substantia nigra pars compacta *(SNpc) are particularly sensitive to injury because they have mitochondrial defects [7], reduced anti-oxidant capacity (such as low reduced glutathione levels), and high content of dopamine, melanin and lipids, which are all prone to oxidation [4]. In addition, the SN is particularly rich in microglia [8], which once activated could provide for a potentially damaging environment surrounding the neurons in that area of the brain [4].

To directly test the hypothesis that inflammation in the brain can lead to selective loss of dopaminergic neurons, several groups used lipopolysaccharide (LPS) either *(i) *chronically or acutely infused into the *substantia nigra *[9,10]; or *(ii) *through an *in utero *exposure of developing fetuses to the endotoxin [11]. In the first paradigm, microglia activation preceded dopaminergic loss [9,10] and in the second one selective degeneration of the nigrostriatal dopaminergic pathway was observed in the neonates [11]. These studies provide a potential mechanistic link between inflammation in the brain and dopaminergic neurodegeneration [4]. The LPS models of PD address the central role of inflammation in this disease but they do not distinguish which of the factors produced by activated microglia and astrocytes induce neurodegeneration. This is a very important issue since the mechanisms by which activated glia specifically target dopaminergic neurons remain critical missing links in the proof of a pathogenic role for activated glia in PD.

Prostaglandins are largely produced by activated microglia and reactive astrocytes (less by neurons) in neuroinflammation. Prostaglandins act as potent local regulators of pathogenic processes associated with CNS inflammation. Our new PGJ2-induced PD model supports the hypothesis that localized production of highly reactive and neurotoxic cyclopentenone PGJ2 may be one of such links between activated glia and PD pathogenesis. Herein, we present *in vivo *evidence for a role of PGJ2 in the death of dopaminergic neurons in the SNpc. We also show that in this dopaminergic neuronal population, PGJ2 induces the formation of ubiquitin-protein aggregates containing TH and α-synuclein. Furthermore, PGJ2 leads to microglial and astrocyte activation. This feed-forward cycle of glial activation and neuronal injury could be a driving force for progressive dopaminergic neurodegeneration observed in PD.

## Methods

### Animals

Male FVB mice, 11-week old weighing 33 g to 39 g, were obtained from Taconic Farms, Germantown, NY. Mice were singly housed on a 12-h light/dark cycle with food and water available *ad libitum*. The room temperature was maintained at 23°C and 50–70% humidity. Animals were habituated for two weeks before commencement of surgery, which was initiated at 13 weeks of age. All mice were treated in accordance with the guidelines for Animal Care Use at NIH. All efforts were made to reduce animal suffering and the number of animals used.

**Figure 1:**
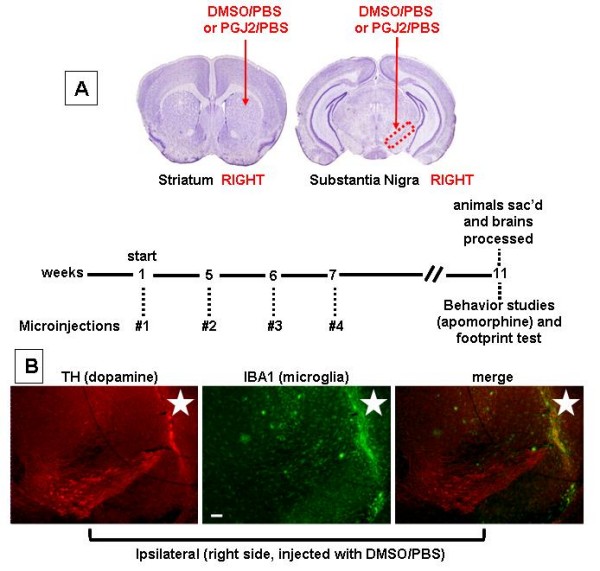
**A. Schematic representation of the experimental design**. Subchronic microinjections into the right *substantia nigra *and *striatum *were processed at 13, 17, 18 and 19 weeks of age. Mice (four groups) were microinjected with either DMSO/PBS (vehicle) or three concentrations of PGJ2/PBS (3.4 μg, 6.7 μg or 16.7 μg) per injection. All animals were perfused intracardially for pathohistological examinations at 23 weeks of age, thus four weeks after the last injection. Behavioral tests were performed four weeks after the last microinjection. **B – Site of injection**. Immunofluorescence analysis with anti-tyrosine hydroxylase (*red*, dopaminergic neuronal marker) and anti-IBA1 (*green*, activated microglia marker) antibodies revealed nonspecific damage at the site of injection in a mouse infused with DMSO/PBS (vehicle). The star indicates the site of injection. Scale bar = 100 μm.

A total of 23 mice received unilateral injections of PGJ2 or vehicle (DMSO) into the striatum and SN simultaneously. The experiments were carried out in four groups of mice. One group received DMSO/PBS (n = 6) and three groups received PGJ2 concentration of 3.4 μg, 6.7 μg or 16.7 μg PGJ2 in DMSO/PBS per injection (n = 6 per group, except the last concentration n = 5), respectively (Fig. 1). Each mouse served as its own control, since there was no surgery on its left-side (contralateral to the lesion). In addition, mice injected with DMSO/PBS served as controls for PGJ2 infusion, since they received unilateral injections of DMSO/PBS solution. Upon microinjection some nonspecific damage (gliosis) was seen at the site of injection (Fig. 1, star indicates site of injection). The injection site is not evident in most of the other figures because they were taken in planes different from the injected one.

DMSO (dimethyl sulfoxide) was the solvent for PGJ2 and its final concentration was 17% for all microinfusions. The solutions were freshly prepared and stored for a maximum of 2 h under cold (4°C) and dark conditions.

### Choice of PGJ2 dose and frequency

PGJ2 was from Cayman Chemical (Ann Arbor, MI). Currently available methods to measure PGJ2 levels in cells or in human tissues and fluids are likely to lead to an underrepresentation of its total amounts formed and will not reflect its biological activity [12]. This is because PGJ2 is highly reactive with thiol-containing intracellular components like glutathione or thiol-containing proteins via Michael addition [13]. It is possible however to estimate the rate of formation of PGJ2 from PGD2 tissue concentrations as PGJ2 is formed from PGD2 by a non-enzymatic first order dehydration reaction. Notably, PGD2 synthesis in mouse brain increased under stress conditions from ~17 pmoles/g brain to ~102 pmoles/g of brain (a six-fold increase) as a result of only a 30 second hypoxia [14]. In addition, microglia PGD2 levels increased 23-fold upon activation with a calcium ionophore [15]. These findings support the view that PGD2 synthesis is extraordinarily dynamic and consequently PGJ2 levels are likely to also increase significantly during inflammation.

Activated astrocytes and microglia make large quantities of prostaglandins such as PGE2 and PGD2 [4] as well as PGJ2 [16]. There is no available means to specifically increase endogenous PGJ2 levels because PGJ2 is spontaneously derived from PGD2 by non-enzymatic dehydration. Thus to assess the effects of increases in PGJ2 we infused it directly into the brain.

In choosing the PGJ2 concentrations for infusion we had to consider that *(1) *the injected pool of PGJ2 would be in equilibrium via active transport [17,18] with the blood circulating through the injected area, and *(2) *that PGJ2 binds very quickly to blood albumin, leading to a continuous removal of PGJ2 from the site of injection. In one study it was estimated in cell cultures that ten percent fetal bovine serum reduces the concentration of PGJ2 available to cells by 150-fold, by directly and reversibly interacting with PGJ2 and sequestering it in the medium [19]. The latter study also demonstrated that human serum had similar PGJ2-sequestration properties. Moreover, due to slight hemorrhage at the site of injection, the released serum albumin will bind some of the injected PGJ2, leading to a further reduction of the available PGJ2. Consequently, the amounts of PGJ2 that diffuse into to the brain tissue contiguous to the site of injection are bound to be significantly lower than those initially infused.

In addition, we wanted to establish a progressive PD model, one in which there is a progressive loss of SNpc dopaminergic neurons. In a recent study, 8 μg of 6-OHDA were bilaterally infused into mouse striatum daily for 5 days [20]. This subchronic paradigm provoked a moderate, but significant loss of nigral neurons while a single injection did not. All of these studies support our choice to infuse the mice unilaterally with three concentrations (3.4 μg, 6.7 μg and 16.7 μg per injection) of PGJ2, similar to the 6-OHDA concentration in the study described above.

All animals received four microinfusions per site of injection at the following ages: initial injections were given at 13 weeks of age followed by individual injections at 17, 18 and 19 weeks of age (Fig. 1). Our reasoning was that by giving a total of four injections per site, we would mimic to a certain extent subchronic inflammation.

### Surgery and "subchronic" microinfusion

Mice were anesthetized with 2.5% isoflurane in oxygen (0.6 L/min flow rate) and placed in a stererotaxic frame (Model 5000 Small Animal Stereotaxic Instrument; Kopf Instruments) fitted with a mouse anesthesia mask (Model 907, David Kopf instruments). A 2 μl Hamilton microinjection syringe (7002 KH) with a 25-gauge needle was attached to the stereotaxic frame via a Universal Holder (David Kopf Instruments). Holes were drilled into the skull and the needle moved to the injection site and inserted slowly. Unilateral (right side) injection sites were determined using a mouse brain atlas [21]. The following coordinates were used relative to bregma: *substantia nigra*, RC -3.25 mm; ML ± 1.25 mm; DV +4.13 mm; *striatum*, RC +0.5 mm; ML ± 2.0; DL +2.5 mm. The needle was left in position for 5-min before two μl of the desired solution were injected into the right side over 10-min (~0.2 μl/min). At the end of the injection, the incision was sutured. The mouse was removed from the stereotaxic apparatus, given a subcutaneous injection of one ml of sterile saline, and left in a warm place to recover. Multiple injections were administered using the same cranial openings drilled at the initial microinjection sites. After each microinjection the incision was sutured and swabbed with betadine solution. Animals were continuously monitored by the staff veterinarian and no signs of infections were noted.

### Immunohistochemistry

For immunohistochemical analysis mice were perfused at 23 weeks of age, thus four weeks after the last injection. Mice were anesthetized using a lethal dose of ketamine/Ace (100/3 mg/kg of body weight, intraperitoneal) and perfused intracardially with 60 ml of Tyrode (RT), followed by fixative (4% paraformaldehyde, 7% saturated picric acid, 0.1% glutaraldehyde in PBS at 4°C). Brains were removed, postfixed for two hours in the same fixative at RT, and cryopreserved in 15% sucrose overnight at 4°C. They were then frozen in dry ice and stored at -80°C until further analysis. Free-floating coronal sections (30 μm thick) from the *substantia nigra *were cut on a sliding microtome HM440E (Microm, Waldorf, Germany).

For immunostaining, sections were permeabilized by sequentially washing with TBS containing 0.5% Triton X-100 (TBS-T)/TBS/TBS-T for 10-min each. Sections were then incubated with the primary antibodies in TBS-T overnight at 4°C and washed again as above. The sections were then incubated for 90-min with the first appropriate labeled secondary antibody (anti-mouse or anti-rabbit antibody, 1:100) in TBS-T and washed again. For sections that were triple stained, tissues were incubated with a second secondary antibody (anti-mouse or anti-rabbit, 1:100) for an additional 90-min, followed by three washes with TBS (10-min each), incubation with Hoechst 33342 (a nuclear stain, 1:500 in TBS) for 30-min and three final washes with TBS. Sections were mounted on glass slides, using 80% glycerol in TBS. Cell staining was visualized with a Leica TCS SP2 confocal microscope (Leica microsystems, Exton, PA). Symmetric areas from injected and control (contralateral, left side) sides as well as DMSO/PBS and PGJ2/PBS injected (ipsilateral) sides were compared.

Primary antibodies: dopaminergic neurons [tyrosine hydroxylase (TH+), 1:500, Abcam (Cambridge, MA), ab6211, rabbit or 1:50, TOHA1.1, mouse, kindly supplied by Dr. C. Cuello, McGuill University, Canada]; neurons [NeuN, 1:50, Chemicon (Temecula, CA), mab377, mouse]; GABAergic neurons [GAD67, 1:1,000, Chemicon (Temecula, CA), mab5406, mouse]; ubiquitin inclusions [ubiquitinated proteins, 1:200, Dako Cytomation (Carpinteria, CA), Z0458, rabbit]; α-synuclein [1:250, BD Transduction (San Jose, CA), 610787, mouse]; microglia [IBA1, 1:500, Wako Chemicals (Richmond, VA), 019–19741, rabbit]; astrocytes [GFAP, 1:500, Sigma-Aldrich (St. Louis, MO) G3893, mouse]. Secondary antibodies: anti-rabbit Alexa 594, A21207, anti-mouse Alexa 488, A21206, and anti-mouse Alexa 350, S11249, all from Molecular Probes, Carslbad, CA and anti-mouse CY3, 1:200, from Amersham Biosciences, Piscataway, NJ.

Cell counts: the number of TH+ cells was determined using a computer-assisted image analysis system, consisting of an Axiophot photomicroscope (Carl Zeiss Vision, Hallbergmoos, Germany) comprising a Zeiss planapochromat 100 × oil objective equipped with a computer-controlled motorized stage, a video camera, and the Stereo Investigator software (MicroBrightField, Williston, VT). The number of TH+ cells was counted in 6–8 sections/mouse (BREGMA coordinates -2.54/-3.64) on the delineated SNpc (ipsilateral, right side). We compared TH+ cell numbers in five DMSO-infused mice with those of five mice infused with 16.7 μg PGJ2 because the latter group of mice showed the greatest degree of change. Means and S.E. for each of the two groups (DMSO and PGJ2-treated) were determined and the *p *value calculated with the two-tailed test.

### Animal behavior

Simple behavioral tests in control and PGJ2-treated mice were performed four weeks after the last injection. Asymmetry in body posture and gait abnormalities were tested with the curling test and the footprint test, respectively. The degree of nigrostriatal damage was assessed with the turning behavior test.

#### 1) Curling

The curling test evaluates any asymmetry in body posture [22]. The mouse was lifted gently two cm above the bedding for 5-sec and any ipsilateral deviation from its vertical body axis of 10° or greater was recorded. The test was repeated three times for each animal.

#### 2) Footprint test

Mice were placed in a 5-cm wide, 85-cm long corridor. The floor of this corridor was covered with white absorbing paper. The animals were first trained to pass straight forward through the corridor. After this training, the paws were colored with different colors (red for the forepaws and black for the hindpaws), and the mice were then placed into the corridor [20]. Step frequency and stride length were determined with the program Footprints version 1.22 [23].

#### 3) Asymmetric circling motor behavior

This behavior is dependent on the degree of nigrostriatal damage. We tested for this behavior upon administration of apomorphine, a dopamine receptor agonist. Rotation is more prominent after apomorphine (APO, Sigma-Aldrich, St. Louis, MO) due to imbalance between lesioned and unlesioned hemispheres [6]. APO leads to a reversal of rodent asymmetry, in that the animals which otherwise have an ipsiversive asymmetry now turn contraversively. Furthermore, the induction of contraversive turning occurs rapidly within minutes after drug administration [24,25]. APO was administered (1.5 mg/kg, intraperitoneally) to reverse animal asymmetry to determine whether this behavior is indeed due to SN damage and not disruption of a different sort of neighboring cell type.

## Results

### PGJ2-microinfused mice exhibited loss of dopaminergic neurons in the SNpc but the GABAergic neurons in the SNpr were spared

**Figure 2:**
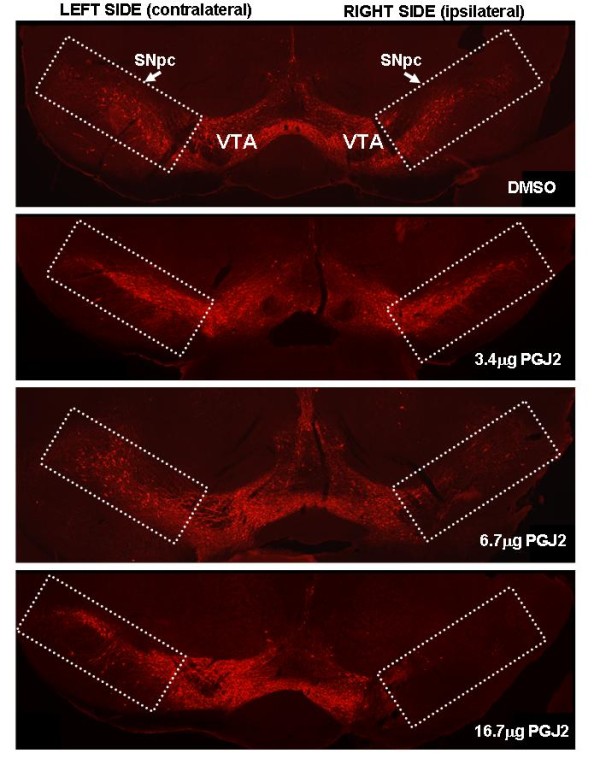
**Representative coronal sections of the ventral midbrain showing TH-immunoreactive neurons**. TH-immunoreactivity was strong in VTA and SNpc of control (DMSO) mice and mice treated with 3.4 μg PGJ2 (*two top panels*), but decreased in a concentration-dependent manner in the right SNpc (ipsilateral to the lesion) of mice treated with 6.7 μg or 16.7 μg of PGJ2 (*two bottom panels*).

**Figure 3:**
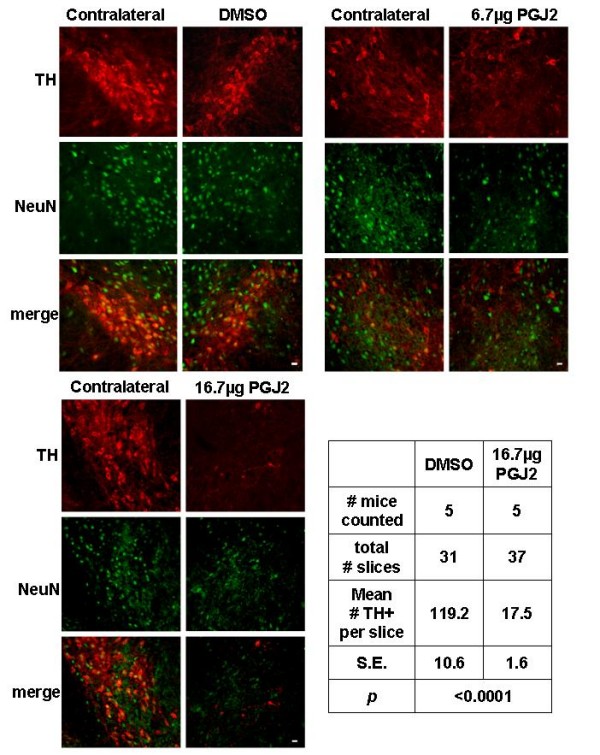
**PGJ2 dose-dependent loss of dopaminergic neurons in the SNpc**. TH (*red*, dopaminergic neurons) and NeuN (*green*, all neurons) immunostaining of SN shows a dose-dependent loss of dopaminergic neurons after microinjections with 6.7 μg or 16.7 μg of PGJ2. No dopaminergic neuronal loss was observed in the side contralateral to the injection site. Scale bar = 20 μm. The table lists TH+ cell counts in ipsilateral SNpc slices of DMSO- and PGJ2(16.7 μg)-infused mice determined as described under Methods.

To assess the vulnerability of nigral dopaminergic neurons to the neurotoxic prostaglandin PGJ2, FVB mice received four injections of PGJ2 at 13, 17, 18 and 19 weeks of age. Mice were processed for immunohistochemical analysis four weeks after the last injection. At 23 weeks of age TH+ staining showed loss of SNpc dopaminergic neurons in a dose-dependent manner (Fig. 2, immunofluorescence staining). Mice infused with 16.7 μg PGJ2 exhibited an estimated 85% decrease in TH+ cells in the ipsilateral SNpc, compared to DMSO-infused mice (see the table in Fig. 3). That the loss of TH staining was due to neuronal loss and not to TH down-regulation is asserted by the PGJ2 concentration-dependent loss of NeuN neuronal staining (Fig. 3).

**Figure 4:**
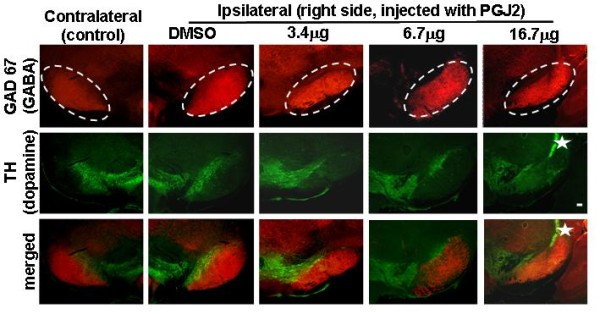
**GABAergic neurons in the SNpr are spared in PGJ2-treated mice**. TH (*green*, dopaminergic) and glutamic acid decarboxylase (GAD-67) staining (*red*, GABAergic) immunostaining of SN shows a dose-dependent loss of dopaminergic but not of GABAergic neurons after microinjections with 6.7 μg or 16.7 μg of PGJ2. The ellipse indicates the SNpr. The star indicates the site of injection. Scale bar = 100 μm.

One of the most challenging aspects of PD is to explain why the dopaminergic neurons in the SNpc are particularly vulnerable to neurodegeneration. One possibility is that the susceptibility to stress conditions is exacerbated by the pro-oxidant properties of dopamine. In fact, we observed that the GABAergic neurons of the *substantia nigra pars reticulata *(SNpr) were spared (Fig. 4) even in mice treated with the highest (16.7 μg) PGJ2-concentration. These data support that SNpc dopaminergic neurons are indeed more sensitive to the neurotoxic effects of PGJ2 than the neighboring GABAergic neurons in the SNpr.

To rule out the possibility that the prostaglandin did not reach the SNpr, we validated our infusion protocol by injecting equivalent amounts of the dyes methylene blue (water soluble) or Sudan black (water insoluble) to mimic the conditions used to inject PGJ2. Upon sectioning, it was clear that both dyes reached the SNpc as well as the SNpr (*not shown*).

### Detection of aggregates with ubiquitinated proteins and α-synuclein in mice treated with PGJ2

**Figure 5:**
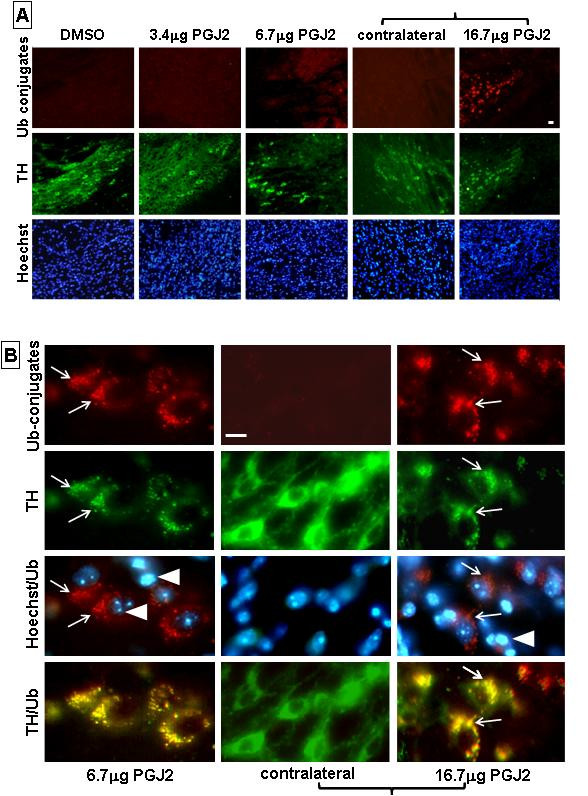
**Aggregates with ubiquitinated proteins are detected upon PGJ2 infusion**. **A & B**. Immunostaining for ubiquitinated proteins (*red*) and TH-positive neurons (*green*) was performed four weeks after the last PGJ2 microinjection. Dopaminergic neurons in the SNpc of mice treated with 6.7 μg or 16.7 μg of PGJ2 exhibited clear aggregates (*arrows*) with ubiquitinated proteins. Nuclear staining (Hoechst) in the mice treated with the two highest doses of PGJ2 also exhibit an apoptotic morphology, i.e. a fragmented and/or condensed appearance (*arrowheads*). No difference was apparent between DMSO-treated mice and mice treated with the lowest PGJ2 concentration (3.4 μg). Panels with brackets depict ipsilateral (infused) and contralateral sections of the same mouse. Scale bar = 20 μm in **A **and 10 μm in **B**.

In mice infused with DMSO and 3.4 μg of PGJ2 only low levels of ubiquitinated proteins were detected. The latter (*stained red*) exhibited a diffuse distribution throughout the cytoplasm of the TH-positive cells (*stained green*) in the SNpc (Fig. 5A). Infusion of 6.7 μg and 16.7 μg of PGJ2 caused the formation of intracellular aggregates of ubiquitinated proteins observed in the few spared TH-positive neurons in the SNpc (Fig. 5A and 5B). TH immunostaining (*green*) co-localized with the ubiquitin aggregates (*red*) suggesting that the enzyme is trapped in the aggregates containing ubiquitinated proteins.

In cells containing protein aggregates (Fig. 5B, *arrows*) nuclei exhibit the typical morphology associated with apoptosis, i.e. a condensed or fragmented appearance (Fig. 5B, *arrowheads*). This nuclear morphology suggests that cells exhibiting protein aggregates are committed to the apoptotic pathway.

**Figure 6:**
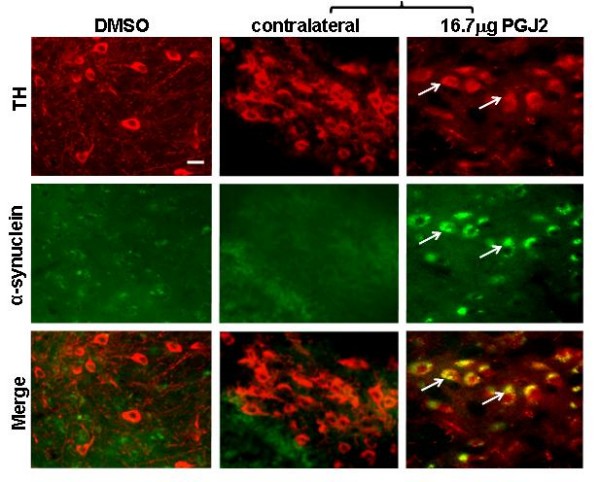
**Aggregates with α-synuclein are detected upon PGJ2 infusion**. Immunostaining for α-synuclein (*green*) and TH-positive neurons (*red*) was performed four weeks after the last PGJ2 microinjection. Dopaminergic neurons in the SNpc of mice treated with 16.7 μg of PGJ2 exhibited clear aggregates (*arrows*) with α-synuclein. Panels with brackets depict ipsilateral (infused) and contralateral sections of the same mouse. Scale bar = 20 μm.

We investigated if PGJ2 infusion causes changes in α-synuclein distribution in the SNpc. Immunostaining with antibodies to α-synuclein (Fig. 6) revealed immunoreactive aggregates in TH+ neurons in SNpc in mice treated with the highest PGJ2 concentration (16.7 μg). This was not surprising, since we previously demonstrated that PGJ2 induces the formation of α-synuclein aggregates in human neuroblastoma SK-N-SH cells [26].

### Increase microglia and astrocyte activation in the SNpc in response to PGJ2

**Figure 7:**
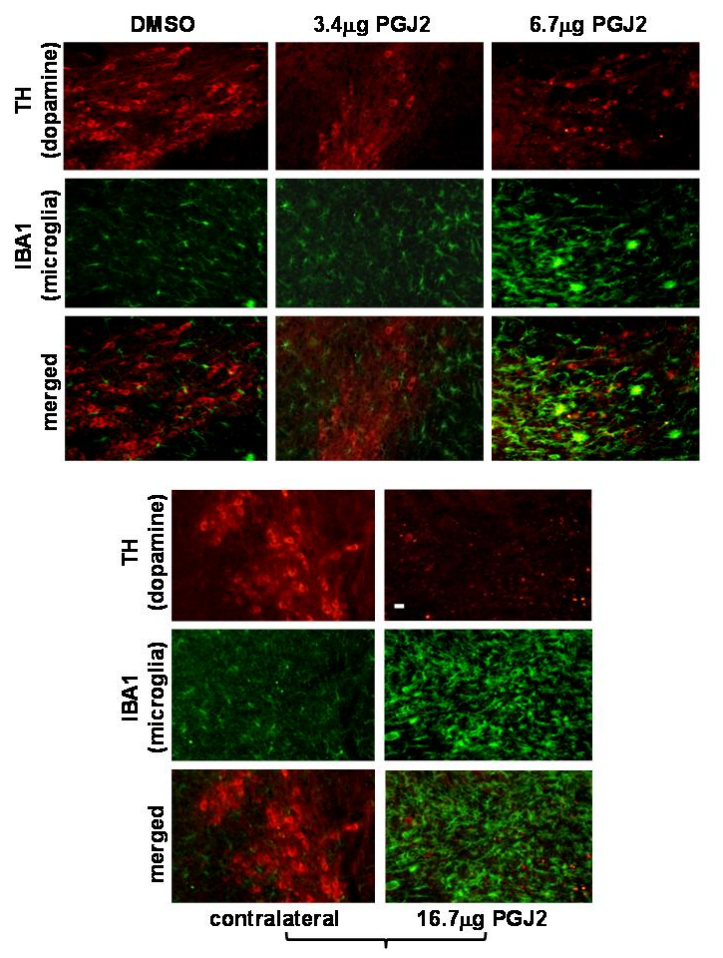
**PGJ2 dose-dependent microglia activation in the SN**. TH (*red*, dopaminergic) and IBA1 (*green*, activated microglia) immunostaining of SN shows a dose-dependent loss of dopaminergic neurons coinciding with microglia activation caused by treatment with the two highest PGJ2 concentrations (6.7 μg or 16.7 μg). No difference was apparent between DMSO-treated mice and mice treated with the lowest PGJ2 concentration (3.4 μg). Panels with brackets depict ipsilateral (infused) and contralateral sections of the same mouse. Scale bar = 20 μm.

**Figure 8:**
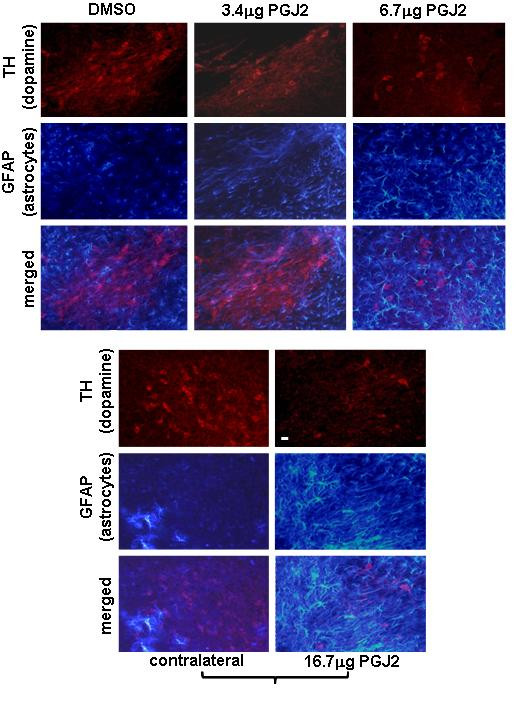
**PGJ2 dose-dependent astrocyte activation in the SN**. TH (*red*, dopaminergic) and glial fibrillary acidic protein (GFAP, *blue*, activated astrocytes) immunostaining of SN shows a dose-dependent loss of dopaminergic neurons concurring with astrocyte activation caused by treatment with the two highest PGJ2 concentrations (6.7 μg or 16.7 μg). No difference was apparent between DMSO-treated mice and mice treated with the lowest PGJ2 concentration (3.4 μg). Panels with brackets depict ipsilateral (infused) and contralateral sections of the same mouse. Scale bar = 20 μm.

Our studies demonstrate that in mice infused with 6.7 μg and 16.7 μg of PGJ2 there is a potent activation of microglia (Fig. 7) as well as astrocytes (Fig. 8).

### Behavioral changes induced by PGJ2

**Figure 9:**
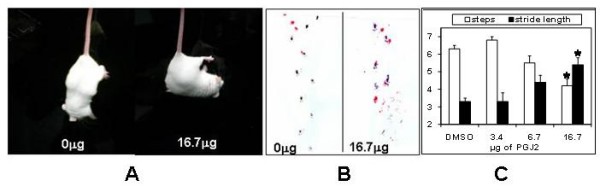
**Symptoms of motor dysfunction in mice treated with the highest PGJ2 concentration (16.7 μg)**. PGJ2 causes severe postural instability (A) and gait impairment (B). The curling test evaluates any asymmetry in body posture. There is a severe deviation from the vertical body axis in PGJ2 treated animals. Representative walking footprint patterns display irregular step pattern, decrease in step frequency and increase in stride length (C, *graph*) in the PGJ2-treated animals. The *asterisk *(*) identifies the values that are significantly different (*p *< 0.01, ANOVA, Tukey-Kramer multiple comparison test) from the control (DMSO).

To address the posture and walking difficulties associated with PD, four weeks after the last injection we performed simple behavioral tests in control and PGJ2-treated mice (Fig. 9). It is clear that mice treated with 16.7 μg PGJ2 exhibited severe postural instability, consistent with a unilateral lesion. All of the mice in this group displayed ipsilateral curling (Fig. 9A). No asymmetry was observed in the other groups. Mice treated with the highest PGJ2 concentration also showed gait impairment assessed by footprint patterns, in particular step frequency and stride length (Fig. 9B and 9C). In addition, mice that were unilaterally microinfused with 16.7 μg of PGJ2 experienced moderate turning behavior (mean 9.0 ± 4.6 rotations/10 min) when administered apomorphine, a DA receptor agonist. Induction of turning occurred within minutes after drug injection. Apomorphine-induced turning was only elicited in the animals that received the highest dose of PGJ2 because they experienced the most substantial lesion to the nigrostriatal pathway. It is well established that with moderate lesions (<80%), no asymmetry occurs [25].

## Discussion

Herein, we demonstrate *in vivo *that infusion of PGJ2 into the *substantia nigra *and *striatum *in mice induces a dose-dependent degeneration of dopaminergic neurons in the SNpc. Due to depletion of dopamine-producing neurons in the basal ganglia, individuals with PD experience deterioration in balance and postural control as well as a progressive reduction in the speed and amplitude of movements. The behavioral tests performed in control and PGJ2-treated mice clearly indicate that PGJ2-treated mice exhibit gait impairment, severe postural instability and moderate turning behavior, the latter upon apomorphine administration supporting a basal ganglia lesion.

Notably, the GABAergic neurons in the neighboring *substantia nigra pars reticulata *(SNpr) were not affected by the PGJ2 microinfusions. It is thus likely that dopaminergic neurons of the SNpc are selectively vulnerable to the toxic actions of PGJ2. At least two reasons can explain why dopaminergic neurons are more susceptible to PGJ2 toxicity. Firstly, PGJ2 up-regulates the expression and increases the activity of COX-2 in cultured neuronal cells [27]. COX-2 readily uses dopamine as an electron donor and in so doing forms the highly cytotoxic dopamine quinone (DAQ) [28]. Secondly, PGJ2 potentiates dopamine toxicity in neuronal cultures by attenuating catechol-*O*-methyltransferase activity [29]. In this way, PGJ2 increases the cytoplasmic availability of dopamine which, in excess of the buffering capacity of the cytosol, will further enhance the production of cytotoxic DAQ. DAQ covalently binds to intracellular proteins, including α-synuclein, leading to the accumulation of pathogenic protofibrils [30].

The ventral tegmental area (VTA), which is in the vicinity of the SN, also contains dopaminergic neurons. However, the VTA dopaminergic neurons were not affected by the PGJ2-treatment, most likely because they are further away from the site of injection and may not be exposed to the toxin. Prostaglandins of the J2 series are highly reactive lipid electrophiles that act as potent local regulators in an autocrine or paracrine manner [31]. Assessment of the susceptibility of the VTA dopaminergic neurons to PGJ2-toxicity may require a similar microinjection paradigm to be applied to this site. The fact that some of the products of inflammation, such as PGJ2, exert their effects in a localized autocrine or paracrine manner could explain why different forms of neurodegenerative disorders, such as AD and PD, share a common mechanism, i.e. neuroinflammation, because the disease form would depend on where in the CNS the pro-inflammatory event takes place. Thus, the variety of disease manifestations associated with neuroinflammation could be correlated with the primary brain region affected by the injurious event, its severity and duration.

Overexpression of α-synuclein increases the risk of sporadic PD, while α-synuclein mutations are associated with inherited PD [32]. Lewy bodies, which are characteristic of PD contain ubiquitinated proteins and also α-synuclein. Distinct aggregates of ubiquitinated proteins and α-synuclein were observed in the few detectable dopaminergic neurons in the SNpc of mice infused with the highest concentration of PGJ2 (16.7 μg). In other PD models such as the acute MPTP and the 6-OHDA models, no inclusions with ubiquitinated proteins were identified (reviewed in [6]), pointing to an advantage of the PGJ2 model. However, continuous MPTP-infusion (treatment for 28 days) also triggered formation of nigral ubiquitin immunoreactive inclusions [33]. It is not surprising that we observed ubiquitin-protein aggregates in mice infused with PGJ2. Data from our laboratory and others clearly demonstrate that PGJ2 impairs the ubiquitin/proteasome pathway (UPP). These endogenous electrophiles *(1) *inhibit ubiquitin isopeptidase activity [34] as well as ubiquitin hydrolases UCH-L1 and UCH-L3 [35], *(2) *induce the formation of cysteine-targeted thyolation of UCH-L1 [36] and of PGJ2/proteasome conjugates [37], *(3) *trigger the oxidation of the S6 ATPase subunit of the 26S proteasome [36], and disrupt 26S proteasome assembly [38]. All of these effects on the UPP promote the build-up of pro-apoptotic and detrimental proteins, such as p53 and ubiquitinated proteins [12]. In fact, cell culture studies demonstrated that PGJ2 induces apoptosis [39-41] and inhibits mitochondrial activity [42-44].

We show that PGJ2 induces microglia and astrocyte activation in the SNpc. Notably, ubiquitin-protein aggregates were observed only in dopaminergic neurons and not in GABAergic neurons nor in glia. Microglia activation is linked to an increase in proteasome activity, which partially explains why microglia are resistant to the large amounts of oxygen free radicals that they produce once activated [45]. This same phenomenon of proteasome activation may protect microglia from the toxic effects of PGJ2. Interestingly, microglia activation is required for their uptake of apoptotic material which is degraded by both the proteasomal and the lysosomal pathways with the proteasome being the major player [46].

The observation that PGJ2-infusion leads to a potent activation of microglia as well as astrocytes, suggests that PGJ2 could be a driving force for progressive dopaminergic neurodegeneration. Although activated microglia and astrocytes release trophic factors, most of their released products are pro-inflammatory and potently cytotoxic [4]. The PGJ2-dependent glial activation may thus be instrumental in further exacerbating the neurodegenerative process observed in PD.

PGJ2 is derived from PGD2, the major prostaglandin produced in the CNS [47,48]. PGD2 is very short lived and readily undergoes *in vivo *and *in vitro *non-enzymatic dehydration to generate the biologically active cyclopentenone J2 prostaglandins, which include PGJ2, Δ12-PGJ2 and 15-deoxy-Δ12,14-PGJ2 (15d-PGJ2) [49]. A noteworthy review suggested that "formation of cyclopentenone eicosanoids [such as PGJ2] in the brain may represent a novel pathogenic mechanism that contributes to many neurodegenerative conditions" [50]. The response to PGJ2 is different from that of agonists that do not form covalent adducts with proteins. A slow steady stream-like release of PGJ2 as a result of chronic neuroinflammation is bound to be cumulative, leading overtime to accumulation of covalent protein adducts with PGJ2 until they reach a toxic threshold. PGJ2 is thus a highly reactive electrophiles that can lead to progressive neurodegeneration [51].

Another product of inflammation, nitric oxide, has a half life of just a few seconds and yet is well accepted as a major signaling molecule in neurons and in the immune system [52]. The same can be said for PGJ2, i.e. that it is a major signaling molecule/oxidative stress agent. Like nitric oxide, PGJ2 has a dual nature: it is protective at low concentrations but toxic at higher concentrations [12]. The synthetic tetrazole HQL-79 is a selective inhibitor of the hematopoietic prostaglandin D2 synthase (H-PGDS) an enzyme that generates PGD2, the precursor of PGJ2. HQL-79 was prepared as an anti-histamine to block the inflammatory signal mediated by both PGD2 receptors, DP1 and DP2 [53]. Selective inhibitors of H-PGDS are more useful to suppress inflammatory reactions than COX-1 and COX-2 inhibitors, because they will not halt production of all PGs, including the cytoprotective and anti-inflammatory ones [53]. These inhibitors do not alter the metabolic flow within the PG cascade, thus do not change the total amount of PGs [53]. Interestingly, HQL-79 suppresses astrogliosis following stab-wounding brain injury [53], suggesting that it could be an excellent anti-inflammatory lead compound against a variety of diseases associated with inflammation.

## Conclusion

We conclude that subchronic microinfusion of PGJ2 into the *substantia nigra*/*striatum *induces pathological features similar to the ones observed in PD neurodegeneration including *(a) *dopaminergic degeneration in the SNpc, *(b) *formation of intracellular aggregates of ubiquitinated proteins and α-synuclein, *(c) *microglia and astrocyte activation and *(d) *behavioral changes. The chemical properties of PGJ2 and its pro-oxidant and UPP disrupting effects render PGJ2 extremely neurotoxic and capable of inducing neuronal cell death [12]. Overall, we propose that the chronic production of neurotoxic products of inflammation (such as PGJ2) will lead to a self-propelling glial (microglia and astrocytes) activation that could be a driving force for progressive dopaminergic neurodegeneration. Over time, this feed-forward cycle of glial activation and neuronal injury would result in sufficient degeneration of the nigrostriatal dopaminergic pathway to lead to the development of symptomatic PD. It cannot be ruled out that glial activation also has neuroprotective effects. Therefore, glial activation may represent a double-edged sword and its role on the neurodegenerative process needs to be further addressed.

## Abbreviations

The abbreviations used are: APO: apomorphine; COX-2: cyclooxygenase-2; DA: dopamine; DAQ: dopamine quinine; DMSO: dimethyl sulfoxide; LPS: lipopolysaccharide; MPTP: 1-methyl 4-phenyl 1,2,3,6-tetrahydropyridine; 6-OHDA: 6-hydroxydopamine; PD: Parkinson's disease; PBS: phosphate buffered saline; PGD2 and J2: prostaglandin D2 and J2: respectively; SEM: standard error of the mean; SNpc and SNpr: *Substantia nigra pars compacta and pars reticulata*: respectively; TH: tyrosine hydroxylase; RT: room temperature; Ub-protein: ubiquitinated protein; UPP: ubiquitin/proteasome pathway; VTA: ventral tegumental area.

## Competing interests

The authors declare that they have no competing interests.

## Authors' contributions

SP and MEFP designed the study. SP performed the animal experiments. MAML contributed to the immunostaining of Figs 2, 3, 4. SP and MEFP contributed to the analysis and interpretation of the data and corrections of the manuscript. MEFP wrote the first draft of the paper. All authors read and approved the final version of this manuscript.
